# Open Science 2.0: Towards a truly collaborative research ecosystem

**DOI:** 10.1371/journal.pbio.3002362

**Published:** 2023-10-19

**Authors:** Robert T. Thibault, Olavo B. Amaral, Felipe Argolo, Anita E. Bandrowski, Alexandra R, Davidson, Natascha I. Drude

**Affiliations:** 1 Meta-Research Innovation Center at Stanford (METRICS), Stanford University, Stanford, California, Unites States of America; 2 Institute of Medical Biochemistry Leopoldo de Meis, Universidade Federal do Rio de Janeiro, Rio de Janeiro, Brazil; 3 Universidade de São Paulo, São Paulo, Brazil; 4 FAIR Data Informatics Lab, Department of Neuroscience, UCSD, San Diego, California, United States of America; 5 SciCrunch Inc., San Diego, California, United States of America; 6 Institute for Evidence-Based Health Care, Bond University, Robina, Australia; 7 Faculty of Health Science and Medicine, Bond University, Robina, Australia; 8 Berlin Institute of Health (BIH) at Charité, BIH QUEST Center for Responsible Research, Berlin, Germany

## Abstract

Conversations about open science have reached the mainstream, yet many open science practices such as data sharing remain uncommon. Our efforts towards openness therefore need to increase in scale and aim for a more ambitious target. We need an ecosystem not only where research outputs are openly shared but also in which transparency permeates the research process from the start and lends itself to more rigorous and collaborative research. To support this vision, this Essay provides an overview of a selection of open science initiatives from the past 2 decades, focusing on methods transparency, scholarly communication, team science, and research culture, and speculates about what the future of open science could look like. It then draws on these examples to provide recommendations for how funders, institutions, journals, regulators, and other stakeholders can create an environment that is ripe for improvement.

## Abstract 2

What might Open Science look like in the future? By examining past initiatives, this Essay offers recommendations to funders, institutions, journals, and stakeholders to create an environment conducive to open science, emphasizing its value in fostering better science.

This article is part of the *PLOS Biology* 20th Anniversary Collection.

## Introduction

The past decades have seen a shift in the nature of human communication. With the advent of the World Wide Web, accessing information from across the globe became commonplace. But it was not until the Web 2.0—also known as the participatory web [1]—that users transformed from passive consumers of information to engaged participants interacting across a dynamic landscape. In a similar vein, the past 20 years have seen information about research become more accessible, through developments like open access and clinical trial registration. More recently, however, open science initiatives are increasingly pushing beyond the goal of simply sharing research products and towards creating a more rigorous research ecosystem. These advancements not only facilitate human collaboration but also enable the development and deployment of automated tools for data synthesis and analysis, which thrive on large quantities of open and high-quality data.

This Essay reviews achievements in open science over the past few decades and outlines a vision for Open Science 2.0, a research environment where the entire scientific process from idea generation to data analysis is openly available. Where researchers seamlessly interact to build on the work of others, and where the research infrastructure and cultural norms have evolved to foster efficient and widespread collaboration. We use this term not simply to suggest a large step forward but to invoke transformational change in the capacity and purpose of a system, as was observed with the Web 2.0.

Realizing this vision requires that we challenge traditional research norms and embrace a collaborative spirit to iteratively improve our research practices and infrastructures. In this sense, we end this Essay with recommendations for how funders, institutions, publishers, regulators, and other stakeholders can foster a research environment that cultivates openness, rigor, and collaboration. We argue for concerted and persistent efforts, supported by sustained public funding mechanisms, that treat open science as a milepost toward a more effective research ecosystem. But first things first: What do we mean by “open science”?

## Open science: A primer

A strict definition for open science has yet to emerge, but most explanations overlap substantially. UNESCO has recently defined open science as “an inclusive construct that combines various movements and practices aiming to make multilingual scientific knowledge openly available, accessible, and reusable for everyone, to increase scientific collaborations and sharing of information for the benefits of science and society, and to open the processes of scientific knowledge creation, evaluation, and communication to societal actors beyond the traditional scientific community.” Increasingly, definitions are extending beyond transparency (e.g., sharing of research outputs) to emphasize its downstream goals (e.g., increased collaboration and greater rigor).

Every step of the research process can benefit from openness, including idea generation, study design, data collection, data analysis, results reporting, and related activities such as grant applications, peer review, and policy development. Openness makes the process and outputs of scientific research more available and easier to evaluate. However, openness by itself does not necessarily imply that research is rigorous, collaborative, efficient, equitable, or conducted with societal priorities in mind. Instead, it allows people to more accurately assess these factors.

Open science is an umbrella term that emerged from several parallel initiatives. Open access aimed to make research publications freely available to the public [2–5]. Open source software and open educational resources strived to dissolve access barriers and foster collaborative communities. Meanwhile, the “replication crisis” reached headlines and catalyzed the uptake of open science as a means to improve the trustworthiness of scientific findings [6–9] (see Box 1 for a first-hand account). Many of these initiatives became possible with widespread adoption of the internet and the ability to share large amounts of information across the globe at low cost. They have now coalesced as a multifaceted movement to open up the research process and its outputs [10].

**Box 1. A personal journey through the reproducibility timescape**

A perspective written by Marcus Munafò, co-founder of the UK Reproducibility Network and Associate Pro Vice Chancellor for Research Culture at the University of Bristol.

My own experience of the problems of reproducibility began early. During my PhD about 25 years ago, I was unable to replicate a key finding that the literature would have me believe was absolutely robust. This was meant to be the foundation of three years of research, and it did not work! It was only because I was fortunate enough to speak to a senior academic who reassured me that the finding was surprisingly flaky that I did not simply decide I was not cut out for a career as an academic scientist. But that knowledge was hidden from view.

More than 20 years later there is far greater awareness of the problem, even if we are still some way from implementing potential solutions. During my postdoctoral career, I started to explore patterns within the published literature such as the decline effect, where the strength of evidence for scientific claims declines over time.

I also saw my own field—the study of genetic associations with complex behavioral phenotypes—transform from what was effectively an enterprise in generating noise (the candidate gene era) to one of collaboration, data and code sharing, statistical stringency, and unprecedented replicability (the genome-wide association era).

Publications such as “Why Most Published Research Findings Are False” [11,12] reassured me that I was not the only one to see the problems, and that they were not unique to any one field. But my various attempts to draw attention to this didn’t make me popular; one senior scientist dubbed me “Dr No”, and later told me he had assumed I was a curmudgeonly 60-year old statistician, rather than a 30-year old psychologist (I took it as a compliment!).

For many years I despaired. Having been talking about the problems for almost 20 years, I have recently found myself focusing much more on potential solutions, and all of the exciting innovations and grassroots enthusiasm for change (particularly among early career researchers). Revolutions happen very slowly, then all at once. Although there is much more to do, it finally feels like we are making progress.

In this Essay, we define Open Science 2.0 as a state in which the research ecosystem meets 2 criteria: the vast majority of research products and processes (i.e., scholarship) are openly available; and scientific actors directly and regularly interact with the openly available scholarship of others to increase research impact and rigor. These collaborative activities would be fostered by appropriate infrastructure, incentives, and cultural norms. These aims appear prominently in recent overviews of open science, including the UNESCO Recommendation on Open Science [10]. We differentiate this state from Open Science 1.0, which we propose as a retronym that meets only the first criteria—widespread openness. We are not implying that current efforts only focus on Open Science 1.0 or that we are close to achieving its more modest goals. Instead, we propose this framework to reflect on how current open science initiatives and cultural norms align with the loftier goals of Open Science 2.0.

### The open science landscape: A whistle-stop tour

Today’s open science initiatives aim to address issues that range from very precise (such as providing nonambiguous identifiers to biological reagents in lab studies) to overarching (like embedding an appreciation for data sharing into a complex research ecosystem). Table 1 outlines 4 distinct topics that demonstrate the diversity of open science initiatives and convey the need for efforts across various fronts. We selected these topics based on our expertise; they are not intended to be exhaustive. Below, we unpack these examples and highlight where some have succeeded and others have fallen short (see also Box 2 for a personal perspective of open science milestones).

**Table 1:** Examples of past developments and future directions in open science.

Topic	Past/ongoing developments	Example initiatives	Future direction
Methods transparency	Adoption of reporting checklists Adoption of persistent unique identifiers for biological reagents	CONSORT guidelines (Consolidated Standards of Reporting Trials) Research Resource Identifiers (RRIDs)	Adoption of standards throughout the research cycle, fostering ongoing quality control
Scholarly communication	Sharing of scholarship beyond journals (preprints, preregistration, data sharing)	Open Science Framework (osf.io) ClinicalTrials.gov arXiv.org	All scholarship shared regardless of “success” Comprehensive linkage of scholarly outputs
Team science	Adoption of contributorship statements Development of large shared databases	Contributor Roles Taxonomy (CRediT) UK Biobank	Employment of diverse specialized scientific roles Flourishing of research teams both large and small
Research culture	Widespread discussions on research culture Coordination of bottom-up efforts to improve research culture	Reproducibility Networks NASA’s Transform to Open Science (TOPS) Declaration on Research Assessment (DORA)	Merging of bottom-up and top-down efforts Incorporation of open science training into curricula Open science as the default

**Box 2. A selection of open science milestones**

A perspective written by Ulrich Dirnagl, Founding Director of the BIH QUEST–Quality, Ethics, Open Science, and Translation Center at BIH at Charité.

Fortunately, the past two decades saw numerous milestones and achievements in opening up science. My selection must therefore be highly selective, almost random from a much larger pool, and certainly biased by personal preference and experience.

I will start in the year 2000 with the publication and endorsement by over 1,000 journals of the ARRIVE guidelines for reporting animal research [13,14]. Although still not uniformly enforced, they were a great leap forward towards making animal research more robust and trustworthy. A must on the list are efforts to limit researchers’ undisclosed flexibility in selecting, analyzing and reporting results as well as fighting publication bias.

Regarding clinical studies, an icebreaker was the creation of the trials registry clinicaltrials.gov by the US National Library of Medicine (2000). A number of initiatives helped shift the emphasis from the results of research to the questions that guide the research and the methods used to answer them: For example, registered reports were proposed in 2012 by Chris Chambers [15], and are now offered as a publishing format by over 300 journals.

No list of milestones would be complete without mentioning the founding of the Center for Open Science (2013), which is currently celebrating “a decade of promoting openness, integrity, and reproducibility of research.” Which brings me to systematic institutional interventions to open up science and change research culture. It will be no surprise that the QUEST Center for Responsible Research, which was established in 2017 [16], features on my list.

Other milestones include reproducibility and multicenter activities such as the Psychological Science Accelerator (2018) [17], or the Reproducibility Project: Cancer Biology, which started in 2013 [8,18].

Finally, I must mention the recent (2022) White House Office of Science and Technology Policy (OSTP) memo [4] to make federally funded research freely available without delay, which I believe will have a tremendous impact on opening up science worldwide.

### Methods transparency

The methods section of many publications lacks key information that would be necessary to repeat an experiment. In response to this lack of transparency, researchers across a range of health disciplines have come together to develop standardized reporting guidelines. The EQUATOR Network (Enhancing the QUAlity and Transparency Of health Research) now includes over 500 reporting guidelines for different types of health research. Some of the highly adopted checklists include CONSORT (Consolidated Standards of Reporting Trials) [19,20], ARRIVE (Animal Research: Reporting of In Vivo Experiments) [13,14], and PRISMA (Preferred Reporting Items for Systematic Reviews and Meta-Analyses) [21]. To achieve their current impact, these guidelines have gone through updates informed by wide-reaching consensus processes. For example, despite the first iteration of the ARRIVE guidelines being endorsed by over a thousand journals [22], they had limited impact on improving transparent reporting, even when authors were explicitly requested to use the ARRIVE checklist [23]. Guidelines were then revised and updated to focus on feasibility and include educational resources and examples. Development of reporting standards is an ongoing process, and some are now being harmonized through initiatives such as the MDAR Checklist (Materials, Design, Analysis, and Reporting) [24,25] and the alignment of guidelines for reporting trial protocols (SPIRIT) and results (CONSORT) [26].

Beyond guidelines that outline what details to include in a publication, research transparency also depends on standardized structures for how to report this information. A few decades ago, catalogs of reagents for biological experiments contained a few hundred listings. A company name and antibody target were generally sufficient to unambiguously identify a reagent. Today, a catalog from a single company can list over 100,000 antibodies, with hundreds of antibodies targeting the same protein. Simply citing a company name and target leaves much ambiguity and, in a surprisingly large percentage of cases, leads scientists to waste money and time trying to optimize the wrong reagent [27–29].

To address the issue, researchers convened meetings and workshops with the editors-in-chief of 25 major neuroscience journals, officers from the US National Institutes of Health (NIH), and representatives of several nonprofit organizations to work on a plan to address the underreporting of reagents. They then proposed a 3-month pilot project in which journals requested that antibodies, organisms, and other tools listed in publications contain the reagent name, catalog or stock number, company name, and Research Resource Identifier (RRID), a reagent identifier that persists regardless of whether companies merge or stock centers move. This RRID initiative [30] is now in its ninth year and over a thousand journals request RRIDs. In 2020, nearly half of published references to antibodies included sufficient information to track the antibody down, a big shift from 15% in the 1990s [31]. By asking researchers to publish RRIDs, researchers were also inadvertently encouraged to double-check their reagents, reducing not only errors in antibodies but also the use of problematic cell lines, with no additional effort on the part of journals [29].

The success of the RRID initiative depended on a dedicated group of volunteers who worked for nearly a decade to overcome an initial unwillingness from actors who held power to make change. The initiative was initially contentious because it added to the workload of journal editors and simply updating author guidelines to request RRIDs proved ineffective. Achieving greater compliance required convincing journals to take an active approach, which depended on the persistence of the RRID Initiative leadership, alongside sufficient infrastructure for authors to easily find their reagents and a helpful helpdesk for when the infrastructure fails to perform as expected. When prominent journals such as *Cell* began to visibly request RRIDs, the conversation shifted. While we could celebrate the success of the RRID initiative as an example of the benefits of grassroots initiatives, an alternative argument can be made: that similar initiatives would be far more common if supported by standard funding mechanisms and greater stakeholder involvement.

### Scholarly communication

Publishing technology has undergone remarkable transformations, and scientists can now instantaneously share nearly all aspects of their scholarship with a worldwide audience. However, the academic research community continues to treat journal articles as the principal way of sharing research and efforts for change generally remain tied to this journal-centric system. One unfortunate legacy of the print era—when publishing was expensive and limited in length and structure—is that publications often serve as an advertisement of research rather than a complete record of the research process and outcomes [32]. This state of affairs, combined with an incentive structure that rewards groundbreaking and positive findings, has led to a muddled scientific record that entails irreproducible studies and wasted resources.

The past few decades, however, have seen several open science initiatives making stepwise progress toward sharing the components of research. These efforts include preregistration of study design and outcome measures, as well as open sharing of materials, protocols, data, and code. Some disciplines have been much more successful than others in these endeavors.

ClinicalTrials.gov and the International Standard Randomised Controlled Trial Number (ISRCTN) were launched in the year 2000 and now contain over half a million registrations. These registries brought transparency to the research process by allowing anyone with access to the internet not only to see what clinical trials were being run but also to have information on the methods, including the study intervention, the inclusion criteria, the outcomes measures of interest, and, increasingly, the results. Their uptake was made possible by funded infrastructure from key organizations such as the US NIH, the European Commission, and the World Health Organization (WHO), and their adoption was fostered by 2 decades of policies from the International Committee of Medical Journal Editors [33], the Declaration of Helsinki [34], and the US Food and Drug Administration (FDA), among others. While the purpose of trial registration was initially to recruit participants and reduce duplication, the infrastructure was iteratively updated. First to make study plans transparent and later to serve as a database of clinical trial results with the aim to reduce selective reporting and wasted research efforts. These updates came with new policies from regulatory agencies, including a requirement for researchers to post their trial results. Notably, policies alone were not enough, and advocacy and external monitoring have been key to press researchers to adhere [35]. Today, most clinical trials are registered and report their results [36–38].

In disciplines beyond clinical trials, preregistration has yet to become standard practice. In psychology, recent estimates for the prevalence of preregistration are lacking, but it likely remains around or below 10% [39,40]. In the social sciences, preregistration prevalence is much lower [41], and in preclinical research, one of the main registries has only 161 registrations as of September 2023 [42–44]. This low prevalence may stem from research protocols in more exploratory fields being less strictly defined in advance as compared to clinical trials. Nevertheless, these disciplines could draw on the experience of clinical trial registration to encourage uptake where applicable and also explore alternative interventions that may prove more viable (e.g., blinded data analysis of electronic health records, as done on OpenSAFELY) [33].

Beyond increasing the uptake of preregistration, we can benefit from ensuring that preregistration is serving its intended purpose. One study found that 2 researchers could only agree on the number of hypotheses in 14% of the preregistrations they assessed [45]. A meta-analysis also found that about one-third of clinical trials published at least 1 primary outcome that was different than what was registered and that these deviations were rarely disclosed [46]. These data underscore the need to acknowledge that, although conversations about preregistration appear to have reached the mainstream, concerted and persistent efforts are needed to ensure their uptake and achieve their intended impacts.

Sharing of research data and code has also recently entered mainstream discussions. At the more advanced end of the spectrum, some manuscripts are now entirely reproducible with a button press [47]. However, a recent meta-analysis of over 2 million publications revealed that while 5% to 11% (95% confidence interval) of publications declared to have publicly available data, only 1% to 3% actually had publicly available data [48]. For code sharing, the estimate was <0.5%. The meta-analysis also found that only declarations of data sharing increased over time. Whether shared data are findable, accessible, interoperable, and reusable (FAIR) is yet another question, and some evidence, at least in the field of psychology, suggests that this is often not the case [49,50]. Meanwhile, several national-level funding agencies are quickly moving towards mandating the open sharing of data (US NIH, Canada’s Tri-Agency). While these policies are a step in the right direction, ensuring their success will take substantial effort beyond the policy alone [51,52].

### Team science

To improve methods transparency and data sharing, we could benefit from employing individuals specialized in these tasks. The predominant model of academic research—where a senior researcher supervises several more junior researchers who each lead almost every aspect of their own project [53]—remains a vestige of an outdated apprenticeship model of scientific research. In practice, each aspect of a research project can benefit from distinct expertise, including domain-specific knowledge (e.g., designing a study), technical capabilities (e.g., statistical analysis), and procedural proficiencies (e.g., data curation and data deposit). Poor distribution of labor and lack of task specialization may be part of the reason data and code sharing remain rare [48,54], publications regularly overlook previous research conducted on the same topic [55], and the majority of studies in some disciplines use sample sizes too small to reasonably answer their research question [56].

Efforts to recognize diverse research contributions are helping usher in a new research model that fosters open science. The Contributor Roles Taxonomy (CRediT), launched in 2014, brings attention to the need for diverse contributions by outlining 14 standardized contributor roles, such as conceptualization, data curation, and writing (review and editing). Dozens of notable publishers have adopted CRediT, and some (e.g., PLOS) require a CRediT statement when submitting a manuscript [57]. While the concept of authorship continues to overshadow “contributorship,” the widespread adoption of CRediT is a first step in recognizing diverse research inputs: including efforts related to open science and reproducibility by including roles in data curation and validation. CRediT statements also provide a dataset that meta-researchers can use to study the research ecosystem and realign incentives [53,58]. The US National Academy of Sciences has taken a step towards this goal by establishing the TACS (Transparency in Author Contributions in Science) website, which will list journals committed to setting authorship standards, defining corresponding authors’ responsibilities, requiring ORCID identifiers, and adopting the CRediT taxonomy.

Promoting role specialization can also help foster the creation of large research teams and, in turn, valuable large-scale research resources. For example, the UK Biobank contains detailed genetic, biological, and questionnaire data from over 500,000 individuals and has been analyzed by over 30,000 researchers in about 100 countries [59–61]. Another initiative, the Brain Imaging Data Structure (BIDS) is a standard for file structure and metadata that allows results from expensive brain imaging studies to be more easily reproduced and meta-analyzed [62]. These efforts, however, require large specialized groups: The UK Biobank includes 15 distinct teams, including imaging, executive, data analyst, laboratory, study administration, and finance [63]; BIDS credits over 250 contributors across 26 roles [64].

Academic funding schemes, however, mainly support small to medium size teams. When larger teams are funded, they generally comprise several smaller teams and sometimes lack the organizational structure and efficiency that specialization can entail, including staff dedicated to human resources, information technology, and project management. Several exceptions exist across the biological sciences where large consortia are becoming more common (e.g., the European Commission Human Brain Project, the US NIH’s Knockout Mouse Program), and in high-energy physics, where CERN has served as a model for large-scale scientific collaboration. Consortia in other disciplines, however, continue to have difficulty securing funding and largely comprise volunteers with their main responsibilities elsewhere (e.g., the Psychological Science Accelerator) [65].

In the absence of mainstream funding opportunities for large and enduring research teams, the possibility of answering certain questions is left to those who can afford it, such as industry, government, and exceptional philanthropists. These actors may not prioritize the advancement of science and betterment of society in the same way one would hope that impartial academics do. For academia to remain competitive across the landscape of research questions, we envision a future where the systems for funding, hiring, and promotion prioritize the flourishing of large and long-lasting research teams.

### Research culture

To embed open science and team science into our research system, we can benefit from considering our research culture*—*the behaviors, expectations, and norms of our research communities [66] (see Box 3 for a personal account). In the absence of a culture that prioritizes openness, tasks like accessing data that support a key finding can remain impossible and sharing your own data can be far from trivial.

**Box 3. The need for a coordinated approach to change research culture**

A perspective written by Fiona Fidler, founding president of the Association for Meta-research and Open Science (AIMOS).

It is almost 20 years since I finished my PhD thesis comparing statistical reform efforts in medicine, psychology and ecology. At that time, I was very focused on why individual researchers didn’t change their practices in light of criticisms, in particular, why null hypothesis significance testing practices did not change in the wake of so many published accounts (literally hundreds) of misuse and misinterpretation.

At that time, many of us thought editorial policy would be a silver bullet. If the editors made the right policies, the researchers would fall in line. How naïve that seems now! What has happened over the past 20 years is recognition of all the other structural and institutional barriers to change. For example, the perverse incentives created by certain metrics and workload models used to assess researcher performance in universities, the evaluations that determine how resources are allocated by funding agencies and so on.

Another big change is the level of coordination in open science reform, for example, the growth of grassroots networks and societies, collective actions, and big team approaches to science. The level of coordination created by organizations like the Society for Improving Psychological Science and initiatives like the Transparency and Openness Promotion guidelines simply did not exist 20 years ago.

Despite increasing awareness of the need for transparent and reproducible research practices, there remains a disconnect between ideals, formal policies, and the actual behavior of researchers. Reproducibility Networks are one example of a collective bottom-up effort to address these gaps. They comprise national consortiums of researchers distributed across universities who can work collaboratively with policy makers from research institutions, government, funders, and the broader research community to drive rigorous and transparent research. First launched in the United Kingdom, Reproducibility Networks now exist in over a dozen countries [67,68]. The UK Reproducibility Network‘s (UKRN) unified voice led to a major strategic investment of £4.5M from Research England to roll out a coordinated effort for training in open science across 18 institutions. UKRN creates a cohesive and consistent message of open science practices that is helping to establish an open science research culture in UK research institutions (e.g., through contributions to parliamentary inquiries [69]).

The Center for Open Science (COS), a nonprofit organization based in the United States, has also been pivotal in advancing open science practices and promoting transparency in research [70]. Many of the COS initiatives, such as the Open Science Framework (OSF), facilitate collaborative and transparent research workflows [71]. Through partnerships, education, and advocacy for open science principles, COS has significantly contributed to the global effort to transform research culture and improve research integrity [72].

To ensure the widespread adoption of transparent and reproducible research, we need a research culture that prioritizes training in open science practices. Training initiatives can be organized at various levels, from individual institutions to international collaborations. Nonprofit organizations (e.g., COS, ASAPbio [73,74]), academic institutions, and funding agencies (e.g., US NIH, Wellcome) provide open science training through initiatives such as curricula integration, professional development programs, funding support, and the provision of resources and workshops to promote open research practices and enhance research quality. These resources teach several topics, including open data, open access publishing, and how to create reproducible research workflows using open source tools like R and GitHub [53]. Emphasizing the importance of open science practices during early career development can be particularly valuable, as it fosters a culture of openness from the outset of a researcher’s career.

However, a general lack of adequate infrastructure and funding poses challenges for establishing and sustaining such initiatives. To overcome these challenges, institutions can support roles dedicated to improving research culture. For example, the University of Bristol in the UK employs an Associate Pro Vice-Chancellor for Research Culture. Making research culture and open science a key part of someone’s job description is likely to foster a better research ecosystem. Additional funding like the Enhancing Research Culture Fund from Research England provides grants to higher-education institutions to implement initiatives for positive research culture [75]. In Germany, the BIH QUEST Center for Responsible Research is a dedicated institutional initiative promoting transparent and reproducible research practices through education, services, tools, and meta-research, with a unique funding structure combining support from the Federal Ministry of Education and Research (BMBF) and the state of Berlin [76–78]. By providing resources and recognition, institutions can create an environment that actively encourages responsible and open research practices.

## A call for Open Science 2.0

Now that we have overviewed a few themes across the open science landscape, let us envision what Open Science 2.0 could look like. We use this term in analogy to the Web 2.0, when the internet shifted from static HTML pages to an interactive forum where people regularly add, develop, and exchange information. Today, we take it for granted that this is the Web. Perhaps in 20 years, researchers will take it for granted that open science always entailed more rigorous, synergistic, and impactful research.

By considering what this ecosystem would look like, we can compare it with the current state-of-affairs to reflect on necessary transitions and paths of least resistance. We argue that an ideal ecosystem-wide implementation of open science would, at a minimum, consist of a modular and dynamic research record, standardization and interoperability, ongoing quality control, and a reorganization of scientific labor. We unpack these terms below.

### A modular and dynamic research record

In Open Science 2.0, researchers would regularly share individual components of their work (such as hypotheses, materials, protocols, data, code, manuscripts, and peer review) once that component is ready for external consumption, instead of at the end of the research cycle. A network of persistent digital object identifiers with citation pathways would link these various digital research outputs and allow other researchers to build upon them. Nondigital components of research (including reagents, researchers, and equipment) would also be given digital identifiers and linked to research outputs, in turn providing a record of their provenance (e.g., RRIDs, ORCIDs). Version control and forking (i.e., independent development of protocols or code based upon previous versions) would assure that relationships to previous items remain transparent while they are dynamically updated. This structure would spur a culture where comments on the work of others, including corrections and suggestions, become an integral part of the research record, instead of being scattered across myriad forums. Such feedback would arrive throughout the research lifecycle and encourage researchers to improve their output’s “record of versions” [79] rather than to defend a static “version of record.”

While this structure may seem fanciful to many researchers, it is already the basis for a thriving community centered around open source software and built upon platforms like GitHub. Within the research ecosystem, protocol repositories such as protocols.io, data repositories such as Figshare, Dryad, and The Dataverse Project, and platforms for sharing individual results such as microPublication apply similar concepts to particular steps of the research process. Nevertheless, these diverse research outputs are still not adequately linked to each other. Organizations such as Octopus and Research Equals provide a way to integrate these different outputs within a single platform, but their uptake remains limited [80].

As modular research outputs become more widely used, they would serve as the main pillar of the research record. Scientific articles would likely continue to exist, but as narrative descriptions of research, rather than the primary account of the research record. In this world, journals would need to make their value clear, as they would no longer be the primary venue for documenting the research record. They could emphasize their role as curators of science, selecting and summarizing the findings they deem most important [81], as evaluators of research through peer review (as exemplified by Peer Community In and *eLife* [82]), or reinvent their services in a multitude of ways [83].

### Standardization and interoperability

For open science to foster collaboration, we can benefit from using agreed upon data structures, vocabularies, and metadata standards that allow both researchers and machines to easily integrate various open datasets and analyze them (i.e., they would be interoperable). Genomics and molecular biology provide strong examples of this standardization and associated interoperability. The creation of large databases such as GenBank and UniProt have led to gene and protein sequence data being deposited in a common format. This standardization fueled a revolution in bioinformatics, allowing large-scale analysis at the touch of a button [84–87]. In a particularly striking example, the Alpha Fold Protein Structure Database used AI to predict structures for over 200 million proteins, most of which were based on UniProt sequences [88]. This is one example of where AI can perform a task infinitely faster, and perhaps better, than any researcher could hope to. Automated tools would also conduct evidence synthesis in near real time and help scientists keep pace with an ever-growing scientific literature. To benefit from the capabilities of AI-powered research, however, requires that data are structured and transparently shared.

For many research fields, shared data still consist of custom-made spreadsheets, in which little attention is paid to standardization. This turns data synthesis into a painful process that can require hundreds of hours of human work in selecting articles on a given question, extracting data and analyzing it. In Open Science 2.0, we envision the level of structured transparency seen in the examples above as being common across disciplines.

### Ongoing quality control

Several open science initiatives promote transparency with the hope that accountability will follow. However, if no person or software is checking the openly shared research outputs, or if openness comes only at the end of the research cycle, the effectiveness of quality control mechanisms remains limited. Historical examples (e.g., in manufacturing [89]) suggest that quality control is much more effective when conducted throughout each stage of a project.

Some initiatives already aim to move quality control earlier in the research process, such as Registered Reports [90]. But these initiatives are still based on what a researcher states they will do or did, rather than an audit of the actual research process. Embedding quality control systems within the routine of academic labs, as is commonplace in many industries, has proved a considerable challenge, and existing initiatives are still at an early stage [91,92]. Leveraging technology to make the research process more open, through the use of open lab notebooks, for example, can allow at least part of this quality control to be distributed and possibly automated. AI tools could warn researchers about missing information, protocol inconsistencies, references to retracted papers, or problematic RRIDs throughout the course of a project. They could also be leveraged during peer review to systematically check for issues that many expert reviewers regularly overlook [93–95] or be applied to entire corpuses of research [96,97]. In Open Science 2.0, we envision widespread transparency in standardized formats that support a mix of automated and manual quality control mechanisms that occur throughout each stage of the research cycle.

### Reorganization of scientific labor

Achieving the level of openness, rigor, and interoperability present in Open Science 2.0 necessarily requires a reorganization of scientific labor to encourage task specialization across larger teams. These teams would include people with roles such as Data Manager, Systematic Reviewer, or Statistician, among others. Beyond teams within an institution, this kind of specialization can also be achieved through open science platforms that allow researchers to interact synergistically. Large-scale, distributed collaborations such as the Psychological Science Accelerator and ManyBabies are open to researchers across the globe who can contribute either with data collection or with other kinds of expertise but currently struggle to acquire sustained funding through standard government grants [65]. Regardless of whether teams are created within or across institutions, those involved in research would be rewarded for their specialization and not be expected to demonstrate proficiencies beyond their specialization. Assessments of research impact would also emphasize large-scale contributions, which would encourage institutions to hire individuals that will bring relevant expertise to existing teams, rather than focusing more narrowly on the potential of single principal investigators.

## A roadmap towards Open Science 2.0

**Fig 1:**
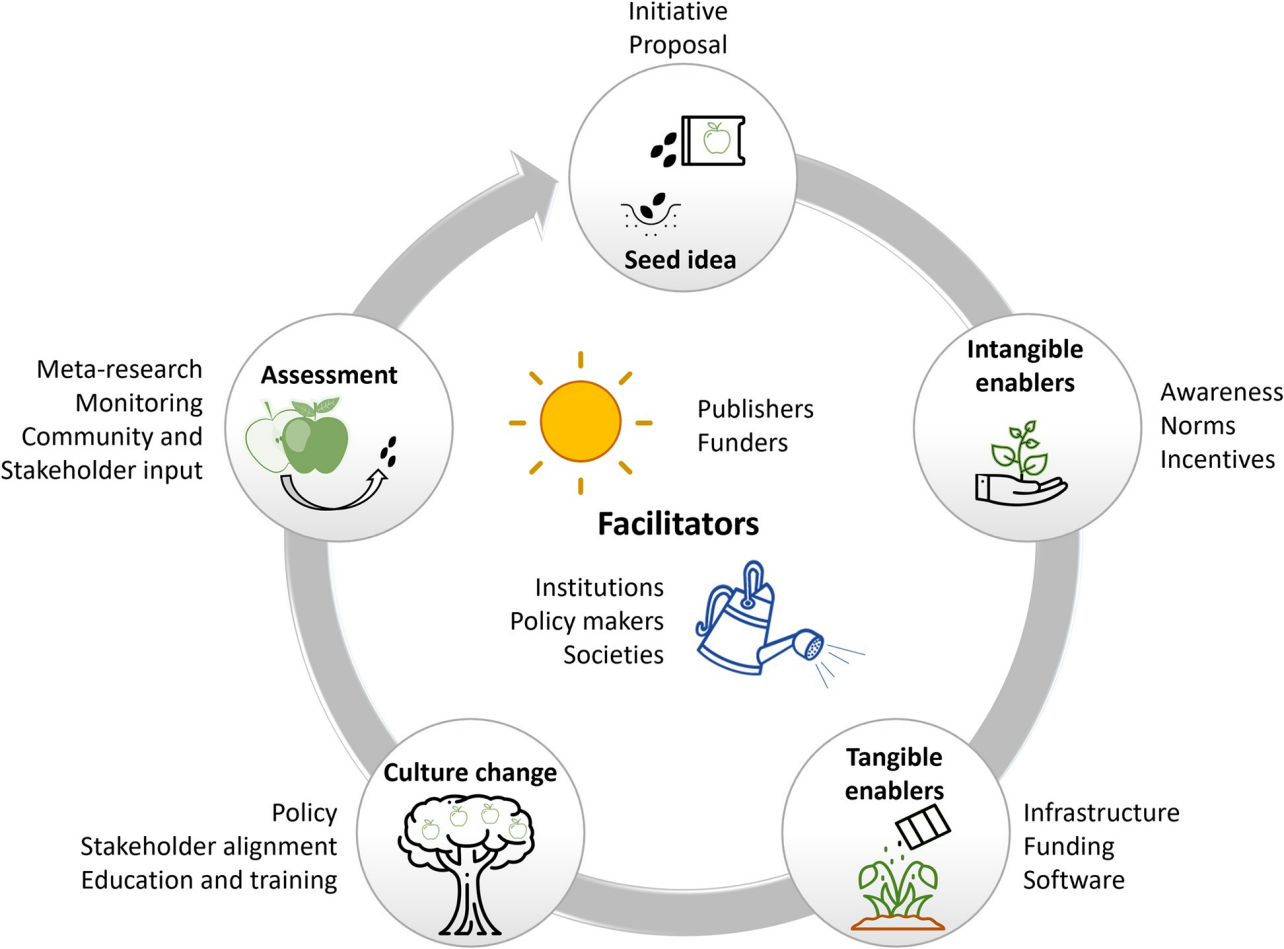
Embedding open science into the research ecosystem requires an iterative process. To move from an open science idea to the realization of that idea depends on enablers and culture change. To ensure open science reforms achieve their intended goals, assessment would be required. The used icons were available under CC-BY4.0.

Drawing on examples outlined earlier in this Essay, we make 7 high-level recommendations for paving the way to Open Science 2.0. These recommendations apply across key stakeholder groups including publishers, funders, institutions, and regulators, among others, who could each enact these recommendations in a variety of ways. We provide specific examples to help readers grasp concrete implementations; however, advocating for specific platforms or workflows goes beyond the scope of the current article. Instead, the recommendations focus on creating an environment where ambitious open science initiatives can flourish and the best solutions emerge (Fig 1).

1. **Monitor progress and policy compliance.** A policy or platform alone is unlikely to spur widespread action if we lack data on progress. Databases like the FDAAA and EU Trials Trackers publicize which institutions are adhering to policies and, in turn, identify targets for improvement. By coupling these trackers with a decade of advocacy and multiple parliamentary inquiries, the UK is now a leader in clinical trials transparency, with results available for over 90% of trials of medicinal products under EU regulations [34]. This success was driven by motivated researchers who pressed regulators to implement their own policies and researchers to adhere. Some institutions take a more proactive approach to monitor their own performance. For example, the QUEST Center for Responsible Research provides a public dashboard reporting on the openness of their research outputs. We recommend stakeholders monitor adherence to their own policies and ideals or provide support for an external body to do so. Progress monitoring would ideally go beyond openness and include measures of rigor and collaboration (e.g., how often datasets are reused [98]).
2. **Fund open science infrastructure, training, and support.** To date, support for open science infrastructure and training has relied in good part on volunteers and philanthropic funding [99]. COS, with more than half a million registered users, was launched with support from Arnold Ventures (previously the Laura and John Arnold Foundation) and continues to depend on philanthropic funding. The RRID Initiative and Reproducibility Networks are largely volunteer driven, in the sense that advancing these initiatives is not part of the job description of most contributors. If the US NIH committed even 0.1% of their total budget to open science initiatives—which represents a very low bar for quality assurance activities across a range of industries—an additional approximately $47M USD [100] would be available to support open science initiatives each year (as proposed by the Good Science Project [101]). Research England has taken note and provided the UKRN with £4.5M for open science training [102], while NASA’s Transform to Open Science (TOPS) Initiative has committed $40M USD over 5 years to accelerate the adoption of open science practices [103].
  Funded infrastructure can also open new opportunities and circumvent downstream costs, like article processing charges and journal subscriptions. For example, the São Paulo Research Foundation (FAPESP), Brazilian National Council for Scientific and Technological Development (CNPq), and Latin American and Caribbean Center on Health Sciences Information (BIREME) launched SciELO (Scientific Electronic Library Online) in 1997. This digital library helped local journals adapt to the online world and now provides infrastructure for over 1,600 open access journals in 17 countries—with most of them being free to publish in and free to read (i.e., diamond open access).
  Hopefully, these types of funding initiatives represent the beginning of a transition to a system where standard government funders take responsibility for ensuring open scientific practices. As a scientific community, we do not rely on volunteers and philanthropists as the primary means to support research; we should not rely on them as the primary means to ensure research is open, rigorous, and collaborative.
3. **Invite innovation.** To discover and implement better practices, organizations must be open to experimentation, or new organizations must emerge. For example, in 2017, the association Peer Community In began a review and recommendation service for preprints that aimed to provide an alternative to journal-mediated peer review. In a similar vein, the journal *eLife* recently decided to no longer make accept/reject decisions and now only reviews manuscripts that are already posted as preprints [83]. If other journals adopted similar policies, all manuscripts would become open access at the time of submission, via the preprint.
  Another project created a publicly available synthetic version of a nationwide database of electronic health records (OpenSAFELY.org). To run an analysis on the real data, researchers must submit their analysis script online, which is logged and made public. This workflow ensures that the analysis is prepared before viewing the data, makes the analysis script publicly available on GitHub, and serves as a form of preregistration. These types of initiatives can be controversial, but that should not be seen as a drawback. If we knew the best methods to address the shortcomings in our research ecosystem, we would already be employing them. To discover which ideas are worth pursuing and which are not, we need journals, funders, institutions, and other academic stakeholders to welcome innovation.
4. **Fund meta-research.** Funding calls for meta-research (research-on-research) remain rare. Researchers have described meta-research as an iterative process that involves identifying problems in the research ecosystem, investigating them, developing solutions, and testing those solutions [104]. Meta-research can be conducted on the scientific landscape as a whole, or on specific organizations and their policies. For example, COS and collaborators developed badges to encourage preregistration and data sharing [105]. They then studied what happened when the journal *Psychological Science* introduced the open data badge and found a substantial increase in the percentage of publications reporting open data [54]. Other researchers then accessed those open datasets and tried to reproduce the results reported in each paper, but had a low rate of success [50]. These authors then suggested performing a reproducibility check during peer review before awarding an open data badge, which could serve as the basis for another interventional study. A similar research cycle has been shown for the badge supporting preregistration [39,55]. Without these meta-research studies, we may end up promoting practices that fail to achieve the ends we desire. In many instances, interventions we hope would work turn out to be administrative burdens with negligible benefits [23,56,106]. Publishers, funders, institutions, regulators, and learned societies could all dedicate funding to internal and external teams to develop their practices, adopt practices used in other disciplines, and test whether they work as intended [107]. Otherwise, we are left guessing what to implement and whether it works.
5. **Align incentives across stakeholders.** Researchers, institutions, funders, publishers, and other stakeholders theoretically share the same end-goals: advancing knowledge and improving the world. Their near-term objectives and incentives, however, can diverge substantially. Academics want to earn a professorship, universities want to score high in league tables, and journals want to increase their impact factor. Initiatives such as Registered Report Funding Partnerships (RRFPs) [108] aim to align these stakeholders and have been encouraged by Reproducibility Networks. They consist of a funder–journal partnership that peer reviews a project’s methodology and, if they agree on its value, provide funding to conduct the study and a guarantee of publication regardless of the results. These types of initiatives, which address the concerns of multiple stakeholders at the same time, may prove more fruitful and harmonious than mandates alone.
6. **Promote teams and specialization**. If everyone at a company was trying to become CEO of their own company, operations would not run smoothly. But this is largely what happens in academia. Many postgraduate students, postdoctoral researchers, and professors all aim to run their own lab, and this desire is built into the academic system (e.g., via professorship tenure). Some research assessment exercises now challenge this system. The UK Research Excellence Framework (REF), an evaluation exercise to determine the distribution allocation of £2 billion to higher education institutions, previously assessed individual research staff within an institution [109]. For the next REF cycle, research outputs will be evaluated at the level of entire disciplines within a university. This structure may amplify the importance of non-research staff and incentivize all actors to engage in collaborative pursuits [110,111]. The Netherlands is also diversifying their assessment criteria to include a range of qualitative and quantitative criteria, including open science, team science, and societal relevance. A structure where diverse roles like statistician and data curator come with the same prestige and salary as a professorship could prove beneficial. Beyond academia, researchers have drawn on examples as diverse as professional sports and animal husbandry to demonstrate the collective improvement when evaluating performance at the group level [112]. By changing the level of selection, openness and cumulative impact can increase.
7. **Treat open science as a means, not an end.** We have seen a sticker that states “Open Science: Just Science Done Right” [113]. We would argue, however, that openness is necessary but not sufficient to do science right. A researcher could run a poorly designed study, draw unreasonable conclusions, and, at the same time, make every aspect of their study openly available. Without quality control mechanisms and an ecosystem where researchers directly build on the scholarly outputs of others, openness may do little to improve the quality and impact of scientific research. For these reasons, we feel it is important to aim for Open Science 2.0, even if practices like data sharing are currently uncommon. If researchers and other stakeholders commit substantial resources to make science open, but research quality, efficiency, and collaboration do not improve, then we risk halting current momentum and lending credence to open science as a box ticking exercise. Taken together, rigor, real-world impact, and collaboration should be considered alongside openness when implementing all of the 6 previous recommendations.

These recommendations aim to cultivate a research ecosystem equipped to handle the challenges and uncertainties of transitioning to Open Science 2.0 and thus avoid unintended consequences. By encouraging researchers to share all their outputs, sharing those outputs in smaller modules, and removing barriers to sharing these outputs, we can expect a vastly larger body of literature; particularly if the evaluation of researchers continues to rely largely on quantitative measures of output. Managing noise and adequately curating and synthesizing data, thus, must remain a concomitant priority.

We also anticipate some degree of upheaval in terms of how credit will be allocated to those involved in research: The units of output will be more diverse and their collaborative nature renders individual contribution more difficult to disentangle. Ideally, an Open Science 2.0 entails an adaptive ecosystem with people and funding dedicated to iteratively addressing challenges as they arise.

Open Science 2.0 could also amplify existing inequalities in scientific research [114]. Large open datasets are more likely to come from the Global North, may prioritize research questions from these populations, and can have limited generalizability (e.g., the overrepresentation of European ancestry in genomic studies [115]). Requiring high levels of openness and rigor could also increase the upfront cost of science. It could risk excluding researchers in the Global South from participating in some circles of scientists and encourage them to analyze open datasets from the Global North rather than front the costs of data collection. Monitoring, funding, and innovation would be necessary to ensure that open science serves people across the globe [116].

Finally, the scale of the challenges to achieve widespread openness in research, and to enact the 7 aforementioned recommendations, should not be underestimated. To illustrate this point, we can reflect on the progress made in open access, which open science proponents, funders, regulators, and publishers have been working on for more than 2 decades. Compared to the full spectrum of Open Science 2.0, or even Open Science 1.0, open access is a relatively simple challenge; authors simply need to upload their submitted or accepted manuscripts to a repository. Organized discussions about open access go back as far as 1995 [117], followed by the Budapest Open Access Initiative in 2001, and mandates for open access from several government funders over the past 20 years. Yet, high-end estimates place the percentage of open access publications around 50% [118], and the high cost of publishing was not addressed but instead transitioned in part from subscriptions to article processing charges. Moreover, because this transition was not accompanied by widespread changes in how researchers are assessed—where research volume remains a priority—other problems such as predatory journals and paper mills emerged. This story highlights the level of persistence and coordination needed to drive change and address unintended consequences. To achieve a research ecosystem that is substantially more open, rigorous, and collaborative will require much larger efforts, supported by sustained funding from governments and institutions.

## Conclusions

The past 2 decades have seen a surge in awareness about open science, with several successful initiatives yielding improvements in particular areas. Yet, transitioning to a research ecosystem where open science practices are the default will require more widespread systemic change. Just as telling individuals to consume less energy is far from sufficient to address the climate crisis; simply asking researchers to make all their scholarship available is unlikely to usher in widespread and collaborative openness. We need concerted and persistent efforts, funded through public mechanisms, and supported by a common understanding of the importance of openness, rigor, and collaboration. Otherwise, we risk underresourcing efforts and falling short of what our communal scientific enterprise could achieve.

## Abbreviations

ARRIVE: Animal Research Reporting of In Vivo Experiments
BIDS: Brain Imaging Data Structure
CONSORT: Consolidated Standards of Reporting Trials
COS: Center for Open Science
EQUATOR: Enhancing the QUAlity and Transparency Of health Research
FAIR: findable, accessible, interoperable, and reusable
FDA: Food and Drug Administration
ISRCTN: International Standard Randomised Controlled Trial Number
MDAR: Materials, Design, Analysis, and Reporting
NIH: National Institutes of Health
OSF: Open Science Framework
OSTP: Office of Science and Technology Policy
PRISMA: Preferred Reporting Items for Systematic Reviews and Meta-Analyses
REF: Research Excellence Framework
RRFP: Registered Report Funding Partnership
RRID: Research Resource Identifier
SciELO: Scientific Electronic Library Online
TACS: Transparency in Author Contributions in Science
TOPS: Transform to Open Science
UKRN: UK Reproducibility Network
WHO: World Health Organization
